# The Acceptance of Key Public Health Interventions by the Polish Population Is Related to Health Literacy, But Not eHealth Literacy

**DOI:** 10.3390/ijerph17155459

**Published:** 2020-07-29

**Authors:** Mariusz Duplaga

**Affiliations:** Department of Health Promotion and e-Health, Institute of Public Health, Faculty of Health Sciences, Jagiellonian University Medical College, Grzegórzecka Str. 20, 31-531 Krakow, Poland; mariusz.duplaga@uj.edu.pl

**Keywords:** sugar tax, vaccination, health literacy, eHealth literacy

## Abstract

*Background*: Public health and health promotion rely on many different interventions, which range from health education and communication, through community mobilisation and changes to environmental conditions, to legal and fiscal actions. The introduction of the increased tax on sugar-sweetened beverages (SSB), popularly called sugar tax (ST), and a mandatory programme of vaccinations are the strategies inciting the most vivid discussions in Polish society. The study was intended to assess the determinants of the attitudes of Polish society regarding the ST and to vaccinations. *Methods*: For the analysis, the data originating from the survey of a representative adult sample of Polish society (*n* = 1000) was used. The survey was based on computer-assisted telephone interviewing (CATI). The assessment of the relationships between the selected variables and the opinions about the introduction of the ST and the safety and effectiveness of vaccinations were carried out using the chi^2^ test and univariate logistic regression models. *Results*: The acceptance of the ST and vaccination showed a significant relationship to the level of health literacy (HL) but not to eHealth literacy (eHL). Respondents having a higher rather than lower HL; older rather than younger; married rather than singles; retired, or on a disability pension, rather than vocationally active and nonusers of the Internet rather than users were more likely to show an acceptance for both interventions. Those more frequently using health care services and those with chronic diseases showed a greater belief in the safety and effectiveness of vaccinations. *Conclusions*: The relationship between the opinions of the two public health interventions analysed and the sociodemographic variables demonstrated similar patterns. Interestingly, the opinions were associated only with HL and not with eHL and users of the Internet were more sceptical about the interventions.

## 1. Introduction

The concept of health literacy (HL) is of crucial importance for health promotion. The definition of health literacy proposed by the World Health Organisation (WHO) belongs to the most popular [1]. It is clear that a focus on the ability to access and use health-related information is essential, but these are not the only aspects of health literacy. The definition proposed by the WHO puts the emphasis, not only on the cognitive, but also on social skills. The context of HL is usually associated with the readiness of people to safeguard their health and to manage their contacts with the health care system. There is growing evidence that insufficient HL may be associated with many unfavourable effects. These include displaying unhealthy behaviours [2,3], lower attention to preventive actions [4], lower knowledge about the disease, not following the physician’s recommendations and limited understanding of the treatment regimen [5,6,7], worse control of the disease [8], and even, a higher risk of hospitalisation and mortality [9]. Some authors also indicated that the lower HL of people is associated with higher expenditure on health care [10].

The concept of digital HL or eHealth literacy (eHL) is used in parallel. It is related to the accessing, understanding, appraisal and application of health-related information available from digital resources [11]. Considering that the Internet is currently one of the primary sources of health information, the role of eHL seems to be obvious; however, the relationship between HL and eHL is not entirely clear. According to Norman and Skinner, HL is one of the types of literacy needed for developing eHL [11]. The correlation between both types of literacy, as substantiated in some studies, is at a level of 0.4 [12,13]. However, the association between eHL and health behaviours or clinical outcomes is not so well documented as it is for HL.

Some authors emphasise a broader meaning of HL going beyond the individual context. According to Baur, a health literate society should be able to create better public health [14]. Such a perception of health literacy which is a precondition of public health actions, resulted in the call to establish the concept of “public health literacy”. According to Freedman et al. (2009), individuals who demonstrate such health literacy, are able to consider and act on health concerns in a community context [15]. The association between HL and the attitudes to community- or nation-wide public health policies has not been frequently examined.

From the onset, health promotion has been proposed as a doctrine combining a whole array of strategies including, not only the development of individual skills, but also the formation of supportive environments, the mobilisation of the community, reorientation of health care services and the shaping of public health policies [16]. It is evident that health promotion relies on many forms of interventions, even if the role of health education and health communication has been frequently overemphasised. However, it appears that in certain circumstances, educational efforts may provide an inadequate response to public health challenges and governments must, therefore, apply legal and fiscal interventions. In many countries, vaccination programmes are mandatory [17]. The taxes or duties imposed on alcohol and tobacco products remain one of the most obvious examples of fiscal measures intended to moderate their consumption [18]. In the last decade, the tax applied to products with a high sugar content became a favoured tool to reduce the harmful effect of sugar-sweetened beverages (SSB) on obesity [19].

The immunisation schedule requires mandatory vaccinations against tuberculosis, hepatitis B, diphtheria, tetanus, pertussis, poliomyelitis, Haemophilus influenzae type b, pneumococci, measles, mumps and rubella in Poland [20]. The National Institute of Public Health’s 2018 Annual Report indicated that depending on the voivodeship, 87.3% to 96.4% of children aged three had been vaccinated against measles, mumps and rubella. However, between 2012 and 2018, the number of Polish parents who refused to accept the vaccination programmes available to their children has increased significantly, from 5340 to 48,609 [21]. This is commonly associated with the influence of antivaccination movements that incite doubts about the safety and effectiveness of vaccines [22]. In 2019, after a discussion lasting several years, the government prepared legislation for a special tax to be imposed on SSB in response to the growing rates of obesity in Polish society. To date, no research has been undertaken to find if HL may be linked to the acceptance of such public health interventions which have triggered significant public debate. The main aim of this study was to assess the association between HL and eHL with the opinions about vaccinations and the introduction of the ST held by a representative sample of the adult Polish population. The role of other variables, including the utilisation of health care resources, the use of information technologies and the sociodemographic characteristics were also analysed.

## 2. Materials and Methods 

### 2.1. Survey

The analysis was based on the data obtained from a survey carried out on a representative sample of the adult Polish population (*n* = 1000). The participants of the survey were recruited by the Biostat Company (Biostat Sp. z o.o., Rybnik, Poland) which has extensive experience in conducting opinion polls [23]. The survey was undertaken using the computer-assisted telephone interviewing (CATI) technique and was completed in one week in mid-December 2016. The sample group was selected by the stratified proportional sampling of the database of mobile and stationary phone numbers developed by the Biostat Company. The survey was carried out with a 58-item questionnaire, including a 16-item short version of the Health Literacy Survey Questionnaire (HLS-EU-Q16) [24]; an 8-item Polish version of the eHealth Literacy Scale (Pl-eHEALS) [25,26] and a set of the items asking about the utilisation of health care resources; health status; the use of the Internet; opinions on public health interventions and sociodemographic characteristics. More details on the sampling procedure and the structure of the questionnaire is available elsewhere [3].

### 2.2. Statistical Analysis

Statistical analysis was performed with IBM SPSS v.24 software (IBM Corp. Armonk, NY, USA). Descriptive statistics were calculated for the variables used in the analysis; absolute and relative frequencies for categorical variables and mean and standard deviation for continuous variables. Chi2 test and univariate logistic regression models were used to assess the association between variables reflecting the opinions about vaccinations and the introduction of the ST as well as potential determinants. In the case of continuous variables, the differences between categories were assessed with either the Student’s *t*-test or the U Mann–Whitney test, depending on the distribution of the variable. For independent variables used in the univariate logistic regression models, odds ratio (OR) and 95% confidence intervals (95%) were calculated. 

### 2.3. Variables

The dependent variables used in the logistic regression were developed after dichotomisation of the two items asking respondents for their opinions about (1) the safety and effectiveness of vaccination, and (2) the introduction of the sugar tax. The initial responses to these items were ranked on a 5-point Likert scale from “I decidedly agree” to “I decidedly do not agree” with a neutral option in the middle. The responses “I decidedly agree” and “I agree” were coded as “1”, other answers as “0”. 

Independent variables used in the logistic regression models included the sociodemographic variables (sex, age, level of education, place of residence, net household income, marital status and vocational activity), the utilisation of health care services (visits to health care facilities, hospitalisations), health status (self-assessed health status, the prevalence of chronic diseases), the use of information technologies (IT; Internet and smartphone use), health literacy (HL) and e-health literacy (eHL). The HL score was calculated according to the guidelines given in the European Health Literacy Survey project [24]. The total score was calculated only if there were at least 14 meaningful responses to the individual questions. The response options “very difficult” and “difficult “were assigned with value “0” and “easy” and “very easy” with value “1”. The total score ranged from 0–16 [3,24]. The eHL score was calculated as the sum of individual scores after assigning values from 1 to 5 to the response options (from “decidedly not” to “decidedly yes”). The minimum total eHEALS that could be achieved was 0 and the maximum was 40.

Respondents filled the questionnaire anonymously after obtaining the information about the study and confirming they agree to participate. The study was conducted in accordance with the Declaration of Helsinki, and the protocol was approved by the Bioethical Committee at Jagiellonian University (No. 122.6120.313.2016 from November 24, 2016). 

## 3. Results

### 3.1. Characteristics of the Study Group

The characteristic of the study group is shown in Table 1. Its sociodemographic structure corresponds with that of the general population at the same time. The mean age was 45.87 (16.16). An HL score could be calculated for the 842 respondents; the mean value (standard deviation, SD) was 12.99 (3.11). The eHL score was calculated only for Internet users (*n* = 849) as 28.91 (5.36). Furthermore, 37.3% of the respondents were convinced that the introduction of the ST was an appropriate measure to reduce obesity in society, 22.6% were undecided and 40.2% did not agree. In turn, 64.4% of respondents believed that vaccines are safe and effective for preventing infectious diseases, 23.1% were unsure, and only 12.5% expressed a negative opinion.

### 3.2. The Opinion about the Safety and Effectiveness of Vaccinations

The respondents convinced of the safety and effectiveness of vaccinations achieved higher HL scores than those expressing the opposite opinion (mean (SD), 13.15 (3.03) vs. 12.70 (3.24), U Mann–Whitney test, *p* = 0.046). In the univariate logistic model, an increase of HL score of one point was associated with a 5% increase in the probability of a positive opinion (OR, 95% CI: 1.05, 1.001–1.10). The opinion was not related to the eHL score (OR, 95% CI: 0.99, 0.97–1.01). The results of chi^2^ tests and univariate logistic regression modelling for the opinion about vaccination as a dependent variable are presented in Table 2. 

Among sociodemographic variables, there was a significant association between the opinion and age, marital status and vocational status. Older respondents were more convinced about the safety and effectiveness of vaccinations (mean age (SD): 48.20 (15.68) vs. 41.67 (16.18), Student’s *t*-test, *p* < 0.001). With every year of age, there was a 3% increase in positive opinions about vaccinations (OR, 95% CI: 1.03, 1.02–1.04). Married persons were more than two times more likely to appreciate vaccinations than singles (OR, 95% CI: 2.23, 1.67–2.27) and widowed persons, divorced or separated nearly 2.5 times (OR, 95% CI: 2.45, 1.57–3.82). As for the vocational status, the employees of public or private entities were less likely to have a positive opinion than those on retirement or those receiving a disability pension (OR, 95% CI: 1.76, 1.27–2.44) but more likely than university students or pupils (OR, 95% CI: 0.54, 0.34–0.86). The analysis based on the chi2 test has not shown any association between the opinions about vaccinations and the place of residence. Nevertheless, the univariate regression model confirmed that respondents living in urban areas with a population of 100,000–200,000, were less convinced about the safety and effectiveness of vaccinations than those living in rural areas (OR, 95% CI: 0.60, 0.39–0.92). 

The opinion expressed about vaccinations was also associated with the number of visits to health care institutions in the preceding year. Those that had to make visits most frequently in the preceding year (at least six or more times) were nearly twice as likely to express a positive opinion about vaccinations (OR, 95% CI: 1.86, 1.22–2.83). A positive opinion was also associated with a higher prevalence of chronic diseases and with an unsatisfactory self-assessment of health status. The respondents who suffered from one or more chronic diseases were more inclined to appreciate vaccinations (OR, 95% CI: 1.44, 1.07–1.96 and 1.63, 1.12–2.36, respectively). The persons who assessed their health status as very good or perfect were nearly 50% less likely to express a positive opinion than persons unsatisfied with their health (OR, 95% CI: 0.55, 0.33–0.91). The users of both the Internet and smartphones were less positive about vaccinations (OR, 95% CI: 0.56, 0.38–0.83 and 0.48, 0.27–0.84). 

### 3.3. The Attitude towards the Introduction of the Sugar Tax

There was a statistically significant association between the HL score and the attitude towards the introduction of the sugar tax. With an increase of the HL score by one point, the probability of a positive opinion increased by 8% (OR, 95% CI: 1.08, 1.03–1.13; Table 3). In turn, there was no significant association between the eHL score and this opinion (OR, 95% CI: 1.01, 0.99–1.03). 

The opinion about the ST showed a similar pattern of the associations with sociodemographic factors as with the opinions about vaccinations. Older persons were more likely to be positive about the ST (OR, 95% CI: 1.02, 1.01–1.03). Singles were less inclined to express a positive opinion than married persons (OR, 95% CI: 1.89, 1.39–2.56) widowed, divorced or separated persons (OR, 95% CI: 1.84, 1.19–2.84). Retired persons, or on a disability pension, were more in favour of the sugar tax than employees (OR, 95% CI: 1.41, 1.04–1.91) but students and pupils were less in favour (OR, 95% CI: 0.41, 0.23–0.73).

There was no association between the variables reflecting the utilisation of health care services and the opinion about the introduction of the ST. Interestingly, the highest acceptance was shown by the persons assessing their health as satisfactory (45.0%) and the lowest by those assessing it as very good or perfect (29.2%) or as unsatisfactory (31.6%). The univariate regression model showed that there was a significant difference only for the comparison of persons assessing their health as satisfactory and unsatisfactory (OR, 95% CI: 1.77, 1.08–2.90). Finally, the chi^2^ test indicated a significant association both between the opinion about the ST and the use of the Internet (*p* = 0.007) or a smartphone (*p* = 0.034). The association was maintained for Internet use only in the univariate regression model. Internet users less frequently agreed that vaccines are safe and effective (OR, 95% CI: 0.62, 0.44–0.88). 

## 4. Discussion

In Poland, the majority of the population (64.4%) would appear to believe that vaccination is a safe and effective method of preventing infectious diseases. Only 12.5% of the respondents were sceptical about vaccines. However, only 37% of respondents believed that the introduction of the ST was an appropriate measure to limit the prevalence of obesity, but 40%were of the opposite opinion. The analysis showed that the attitude towards crucial public health interventions depends on a person’s level of HL but not on their eHL. Furthermore, older persons, married people and the retired or receivers of disability pensions more frequently showed acceptance both for the introduction of the ST and vaccinations than, respectively, younger persons, single people and employees. The users of the Internet and smartphones were less inclined to accept such interventions as were those who self-assessed their health as very good or perfect. Persons with chronic disease or those who declared more frequent visits to health care institutions were more likely to appreciate vaccinations, but not the ST.

According to the WHO Working Group on Vaccine Hesitancy, there is a very extensive list of determinants of vaccine hesitancy. These may be divided into three domains: firstly influences arising from historical, sociocultural, environmental, health system/institutional economic and political factors; secondly, influences stemming from the personal perception of a vaccine or the social environment, and finally, issues related directly to vaccines and vaccination [27]. This reported survey was mainly focused on the sociodemographic characteristics, the utilisation of health care services and the use of IT. 

It seems that the general attitude towards vaccination has been rarely researched. Eilers et al. have confirmed that the acceptance for several types of vaccines is higher among persons of 65 years and older than among those aged 50–65 [28]. A study carried out in Italy showed that vaccine hesitancy was associated with perceived economic hardship and actual refusal with a lower level of parental education [29]. Greater age, receiving information on vaccinations from a physician and the higher quality of such information as well as better knowledge about vaccines were associated with a more positive attitude towards vaccination in a mixed group of Polish pupils, students, patients, parents and healthcare professionals [30].

Most studies reporting on the variables related to the opinions of the general public, or specific populations, about vaccinations are focused on particular types of vaccines. Novak et al. analysed the data from the 2016 National Survey of U.S. Adults [31] and assessed the acceptance of influenza vaccination based on actual vaccination rates. They found that the highest rates of acceptance were by non-Hispanic Whites and Blacks and those aged 65 years and older. The systematic review on influenza vaccination in high-income countries carried out by Lucyk et al. showed that higher socioeconomic status assessed based on education, income, social class, occupation and the level of deprivation was associated with higher levels of influenza vaccination [32].

Mat et al. published a systematic review of acceptance factors of pneumococcal vaccination among the adult population [33]. According to these authors, there were three groups of factors influencing acceptance: the provider’s domain, patients’ perception and sociodemographic factors. In some studies, the group of sociodemographic factors, gender and age were reported to show a significant association with the acceptance of vaccination. Higher acceptance was found among women than men and by those aged at least 65 years old. Another study performed in the USA, limited to the population of adults aged 65 or above, revealed that the uptake of the pneumococcal vaccine was lower among: those of black and Hispanic ethnicity, than among non-Hispanic whites; by the poor rather than those with the highest income; among those with a low level of education than among those with at least college education and finally among those living in rural communities or urban inner-city areas, rather than those living in suburban areas [34].

According to the systematic review published by Lopez et al., higher acceptance of human papillomavirus (HPV) vaccine was associated most consistently with female gender and younger age of respondent parent, female gender of the adolescent, higher household income and previous childhood vaccinations [35]. A recent study by Polla et al. revealed that among parents, those who were unmarried were more likely to be hesitant about the importance of HPV vaccination [36]. 

The analysis reported in this paper showed that a higher level of HL was reflected in a higher acceptance of vaccinations. Consistently, according to the systematic review published by Berkman et al. in 2011, low HL was related to a lower probability of accepting influenza immunisations [4]. However, the results of the systematic review focused on the relationship between HL and attitudes towards various types of vaccinations, published by Lorini et al. in 2018 [37], revealed a more complex picture. The authors included only nine studies in their analysis of respondents representing diverse groups; four studies were undertaken on parents of children who received vaccinations, two among adult citizens, one among adults aged 65 years or more, one among females attending college and one among Hispanic females. The studies yielded unequivocal findings, especially in relation to parents’ attitudes. In the study performed in Israel, higher communicative and critical HL of parents was associated with a greater likelihood of not vaccinating their children [38]. In the study among Dutch parents, all respondents were willing to vaccinate their children against rotavirus when the vaccine was supplied within the National Immunization Programme, but only by those with lower levels of education and lower HL when the vaccine was to be provided by the free market [39]. Another study, performed in the USA, did not find a significant association between maternal HL and the immunisation status of children [40]. In the study carried out in India, higher maternal HL was associated with the likelihood of a child receiving the diphtheria-tetanus-pertussis vaccine [41]. In other groups of respondents, the relation between HL and vaccination uptake varied. Higher HL in the USA increased the likelihood of influenza vaccination among older adults [42,43], and HPV vaccination by undergraduate women [44]. Higher HL was also associated with a higher awareness of HPV and the HPV vaccine by adults in the USA. Additionally, in the USA there was no association between the likelihood of influenza vaccination among adult Hispanic women [45] and adults younger than 40 years [43]. The authors of the systematic review concluded that the role of HL in predicting vaccine hesitancy or acceptance is influenced by various factors including the country, people’s age and the type of vaccine [37]. 

Further studies tend to confirm that the relationship between health literacy and the acceptance of vaccinations is not straightforward and depends on the characteristics of the studied group. In 2018, Castro-Sanchez et al. found a significant association between HL measured with the Short Assessment of Health Literacy for Spanish Adults and the Newest Vital Sign in pregnant women and the vaccination rates against influenza and pertussis [46]. Women rejecting the influenza vaccine had higher HL. Recently, Zhang et al. assessed the relation between HL measured with the standard 47-item version of HLS-EU questionnaire and the attitudes towards vaccination in a group of older adults 65 years and greater [47]. They found that lower competencies related to accessing and appraising health information were associated with more significant problems in reaching decisions about vaccination.

The reported study found no significant association between eHL and the acceptance of vaccination, but the use of the Internet and smartphones was related to a lower acceptance. The overview of systematic reviews published in 2018 by Dumit et al. revealed that eHealth interventions and technology might be useful tools for increasing the uptake of immunisations [48]. However, there are few studies which report on a relationship between eHL and the attitudes towards vaccinations. The research performed by Britt et al. on college students, based on the theory of planned behaviour, showed that eHL was positively associated with the intent for HPV vaccination but not with the actual vaccination behaviour [49]. In a later study in a similar group of respondents from 2017, Britt et al. found that eHL was positively associated with beneficial health behaviours identified by the American College Health Association including seeking for the information on vaccinations and also to a smaller degree, undergoing vaccinations, among college students [50]. 

Additionally, in 2017 Aharony and Goldman reported that parents refusing to vaccinate their children had a higher perceived eHL than hesitant parents or those accepting vaccinations. Additionally, they found that nonrefuser parents had the highest knowledge about vaccinations and the parents refusing vaccinations had the least knowledge [51]. In 2020, Mutur published the results of a survey on eHL and motivators for HPV prevention among young adults in Kenya [52]. She found a positive correlation between eHL and HPV knowledge, perceived risk, self-efficacy and response efficacy. The authors of a systematic review on the association between HL, eHL and health outcomes among patients with long-term conditions found only a few studies in which eHL was assessed [53]. None were related to vaccination attitudes or practices. Currently, eHL as gained new momentum due to the COVID-19 pandemic, but it seems that any high expectations related to its impact on fighting misinformation are yet to be confirmed [54].

The acceptance of the ST has been extensively studied in many countries. In the last decade, surveys carried out in Australia showed that about 40% to 70% of the population were in favour of the tax imposed on SSB. In 2012, Morley et al. reported that 69% of the surveyed participants were in favour of a tax on SSB [55]. Parents were more likely than nonparents and respondents with a higher socioeconomic status, rather than those with a lower status supported a tax on soft drinks and unhealthy food. In 2017 the results of a survey about taxation and nutrition labelling as interventions addressing the incidence of childhood obesity were published [56].

Interestingly, only one-third of respondents strongly supported the introduction of the sugar tax, and 40% were equivocal about it. The level of acceptance of an SSB tax among parents was related to the household’s weekly consumption of soft drinks. In 2018, Sainsbury et al. published the results of an online survey on a nationally representative sample of Australian adults which found that 54.5% of the participants supported SSB taxation. The binary logistic regression models showed that women more than men, younger rather than older respondents and those with a University degree rather than those who did not complete high school, supported the introduction of the tax [57]. The acceptance of SSB taxation was also reported by Farrell et al. in 2019. According to this team, 42% of the Australian population were in favour of the tax imposed on SSB and that the greatest opposition to the tax was expressed by the most disadvantaged group [58]. The analysis performed by Miller et al. on the data coming from two surveys: a face-to-face survey conducted in 2014 and CATI survey in 2017, also showed that persons who attained higher levels of education expressed greater support for SSB tax than those with lower levels of education [59]. The acceptance of a sugar tax was frequently much greater if the tax revenue was to be allocated to obesity prevention, subsidies on healthy food or programmes promoting physical activity [59,60]. 

In June 2017, Belanger-Gravel et al. examined separately support for and the perceived effectiveness of public health interventions aimed at the reduction of obesity among 1000 18–64 years old respondents resident in Quebec, Canada [61]. The introduction of the tax on SSB was strongly supported by 32.8% and somewhat less enthusiastically by a further 27.0% of respondents. 56.3% of respondents assessed this intervention as effective.

The survey performed on USA citizens in 2012 by Rivard et al., demonstrated that SSB tax was supported by 36% of respondents [62]. Greater support was expressed by younger respondents, who had attained higher levels of education and those with body mass index (BMI) <30 kg/m^2^. However, the study published in 2014 by Gollust et al. showed that the tax as a strategy to reduce the consumption of SSB was supported by only 22% of adult Americans who responded to an online survey [63]. In 2015, Donaldson et al. reported that support for the SSB tax was expressed by 52% of respondents participating in a telephone-based survey [64]. The support was related to gender, race, political orientation, SSB consumption but not to age, level of education or annual income. In the study performed by Curry et al. on adults in Kansas in 2018, support for a tax on SSB was confirmed by 40% of respondents, and as in the study of Donaldson et al. it was higher for women and supporters of the Democratic party [65]. In this study, younger respondents were significantly more supportive than older people. 

Petrescu et al. in two parallel online surveys compared the acceptability of nudging initiatives aimed at tackling obesity in the UK and USA on samples of 1093 and 1082 respondents, respectively [66]. Taxation intervention was acceptable to 45.5% of respondents from the UK and 40.7% from the USA. In the UK sample, the perceived effectiveness of the intervention was the only significant predictor of the acceptance of taxation. Among respondents from the USA, the acceptance was associated with the perceived effectiveness and the belief that the environment is responsible for obesity. A French survey published by Julia et al. in 2015 showed that an ST was perceived as an important measure in improving the health of the population by nearly 58% of respondents. The support was even higher if the revenues from the tax were to be used for improving the health care system [67]. Contrary to the findings from Australia and the USA, greater support was expressed by the older rather than the younger respondents. Those who reached higher levels of education were also more supportive. 

In 2019, Kwon et al. reported the results of a multi-country survey (Australia, Canada, Mexico, UK, USA) to assess public support for food policies promoting healthy diets [68]. They found that taxes on sugary drinks were supported by 30.0% in the USA to 53.8% in Mexico. If the revenue raised from the tax were to be spent on subsidising health food, the support would increase considerably to 37.2% in the USA to 66.3% in Mexico. An analysis of the determinants in the pooled data from five countries revealed higher support by females than males, older age groups than the youngest groups, and minorities in comparison to the majorities. According to the latest study on public acceptability of an SSB tax in the Netherlands, lower acceptability was associated with a lower educational level, being overweight, moderate or high SSB consumptions and living in a household with adolescents [69]. 

Finally, the systematic review with a meta-analysis based on 20 papers reporting the results of 22 studies, published in 2019 by Eykelenboom et al., showed that 42% of the public supported the SSB tax; 39% accepted it as a measure to reduce obesity, and 66% supported it if the revenue is used for some type of health-improving initiative [70]. 

In Poland, contrary to the findings from other countries the sex of respondents was not associated with the acceptance of the sugar tax as a measure to decrease the prevalence of obesity. In many countries, a significant association between age and support for the ST was reported. In Australia and the USA usually, younger respondents revealed higher acceptance than older people. In surveys performed in other countries, as in Poland, older rather than younger respondents were more supportive of the ST. Finally, in many surveys, again contrary to findings from Poland, the level of education was associated with the support for the ST. The results of the survey reported in this paper have not shown a relationship between these variables. 

In surveys carried out in other countries, HL, eHL and Internet use were not analysed in respect of the attitude towards sugar taxation. As the penetration of the Internet is growing, and in many societies, the number of nonusers is relatively low, many surveys are performed online. Therefore, the use of the Internet is less frequently considered as a determinant of specific health-related behaviours or attitudes. Nonetheless, it may be puzzling why neither HL nor eHL was assessed as a determinant of the acceptance of fiscal interventions to combat the consumption of unhealthy products. It may be related to the fact that HL is still treated more like a construct reflecting an ability to tackle individual health issues than its relevance to the broader public health context. Interestingly, among the positive impacts of the introduction of a public health Product Tax in Hungary, an improvement of HL was reported [71]. 

## 5. Limitations

There are some limitations of this study which need to be considered. Initially, although the sample size is sufficient to reflect general trends in the Polish population, it may be too small to clarify the relationships between specific variables. Decidedly, further surveys on large samples would be needed to explain the importance of potential determinants of the attitudes towards specific public health measures. Furthermore, the survey was undertaken using the technique of CATI which may result in less profound consideration of the issues presented in the questionnaire. Finally, the survey was performed at the moment when it was not clear that the government was considering the introduction of the ST. Therefore, for some respondents, the prospect of such a public health intervention could seem very distant, and others would not fully understand its consequences. As for the question about vaccinations, a more targeted approach to the study group probably would be needed as the opinions of parents to the vaccination of their children may be different from the opinions of nonparents or older persons. 

## 6. Conclusions

The survey performed on the Polish population showed that there are potentially many variables affecting the opinions regarding the introduction of the ST and the safety and effectiveness of vaccinations. Apart from the sociodemographic factors like age, marital status or vocational status, the utilisation of health care services, the self-perception of health or the prevalence of chronic diseases as well as the use of IT should be considered. Interestingly, it transpired that HL, but not eHL, is related to the acceptance of the ST and vaccination. It may also be surprising that Internet users are more sceptical about such public health interventions. The studies focusing on the relationship between HL and the acceptance of immunisations undertaken on various populations, and concerning specific types of vaccines, yielded unequivocal results. It seems that higher HL does not necessarily lead to a higher acceptance of vaccinations. It may strongly depend on the characteristics of the surveyed population. Surprisingly, the literature on an association between HL or eHL and the acceptance of public health policies is very limited. There is a scarcity of studies which analyse the relationship between HL, eHL and the attitudes to the sugar tax. One could expect that higher HL and eHL should result in higher support for health-promoting fiscal interventions.

## Figures and Tables

**Table 1:** Characteristics of the study group.

Variable	Response Categories	Number of Subjects % (*n*)
Sex	Male	47.7 (477)
	Female	52.3 (523)
Education level	Lower than upper secondary	17.9 (179)
	Upper secondary or postsecondary nontertiary	43.5 (435)
	Bachelor’s degree	12.2 (122)
	Masters’ degree or higher	26.4 (264)
Place of residence	Rural	28.3 (283)
	Urban <20,000	13.4 (134)
	Urban from 20,000 to <100,000	25.5 (255)
	Urban from 100,000 to <200,000	13.4 (134)
	Urban from 200,000	19.4 (194)
Marital status	Single	29.0 (290)
	Widowed, divorced or separated	13.0 (130)
	Married	58.0 (580)
Household net monthly income	≤1500 PLN *	23.9 (239)
	1500–2500 PLN	22.8 (228)
	>2500 PLN	40.9 (409)
	Refused to disclose	12.4 (124)
Vocational status	Employee	43.9 (439)
	Self-employed or farmer	10.7 (107)
	Retired or on a disability pension	28.0 (280)
	University or school student	8.4 (84)
	Unemployed	9.0 (90)
Visits to health care institution in the preceding 12 months	Not used	15.6 (156)
	1–2 times	35.0 (350)
	3–5 times	23.2 (232)
	>5 times	24.2 (242)
Admitted to hospital in the preceding 12 months	No	83.6 (836)
	Yes	16.4 (164)
Chronic disease	No	49.8 (498)
	One disease	29.8 (298)
	>1 disease	17.8 (178)
	Difficult to say	2.6 (26)
Self-assessment of health status	Unsatisfactory	9.8 (98)
	Satisfactory	23.8 (238)
	Good	44.8 (448)
	Very good or perfect	21.6 (216)
Internet use	No	15.1 (151)
	Yes	84.9 (849)
The use of mobile telephony	Nonuser	7.4 (74)
	Mobile phone but not a smartphone	28.4 (284)
	Smartphone	64.2 (642)
Introduction of the sugar tax	I decidedly do not agree	16.4 (164)
	I do not agree	23.8 (238)
	Difficult to say	22.6 (226)
	I agree	28.0 (280)
	I decidedly agree	9.2 (92)
Vaccines are safe and effective	I decidedly do not agree	4.5 (45)
	I do not agree	8.0 (80)
	Difficult to say	23.1 (231)
	I agree	47.5 (475)
	I decidedly agree	16.9 (169)

* PLN—current ISO4217 code for Polish zloty.

**Table 2:** The determinants of the opinion about the safety and effectiveness of vaccinations.

Independent Variable	Categories of the Independent Variable	Safety and Effectiveness of Vaccinations		*p*-Value *	OR	95% CI	*p*-Value ^&^
		No	Yes				
Sex	Male ^#^	37.1 (177)	62.9 (300)	0.34	1		
	Female	34.2 (179)	65.8 (344)		0.34	0.88–1.50	0.34
Education level	Lower than upper secondary ^#^	30.7 (55)	69.3 (124)	0.28	1		
	Upper secondary or postsecondary nontertiary	34.7 (151)	65.3 (284)		0.83	0.57–1.21	0.34
	Bachelor’s degree	39.3 (48)	60.7 (74)		0.68	0.42–1.11	0.12
	Masters’ degree or higher	38.6 (102)	61.4 (162)		0.70	0.47–1.05	0.088
Place of residence	Rural ^#^	30.7 (87)	69.3 (196)	0.21	1		
	Urban <20,000	36.6 (49)	63.4 (85)		0.77	0.50–1.19	0.24
	Urban from 20,000 to <100,000	36.1 (92)	63.9 (163)		0.78	0.55–1.13	0.19
	Urban from 100,000 to <200,000	42.5 (57)	57.5 (77)		0.60	0.39–0.92	0.018
	Urban from 200,000	36.6 (71)	63.5 (123)		0.77	0.52–1.13	0.18
Marital status	Single ^#^	49.3 (143)	50.7 (147)	<0.001	1		
	Widowed, divorced or separated	28.5 (37)	71.5 (93)		2.45	1.57–3.82	<0.001
	Married	30.3 (176)	69.7 (404)		2.23	1.67–2.99	<0.001
Household net monthly income	≤1500 PLN ^#^	33.1 (79)	66.9 (160)	0.066	1		
	1500–2500 PLN	30.3 (69)	69.7 (159)		1.14	0.77–1.68	0.52
	>2500 PLN	37.9 (155)	62.1 (254)		0.81	0.58–1.13	0.22
	Refusal	42.7 (53)	57.3 (71)		0.66	0.42–1.03	0.07
Vocational status	Employed ^#^	38.3 (168)	61.7 (271)	<0.001	1		
	Self-employed or farmer	33.6 (36)	66.4 (71)		1.22	0.78–1.91	0.38
	Retired or on a disability pension	26.1 (73)	73.9 (207)		1.76	1.27–2.44	0.001
	University or school student	53.6 (45)	46.4 (39)		0.54	0.34–0.86	0.010
	Unemployed	37.8 (34)	62.2 (56)		1.02	0.64–1.63	0.93
The use services of the health institution in the preceding 12 months	Not used ^#^	43.6 (68)	56.4 (88)	0.034	1		
	1–2 times	36.3 (127)	63.7 (223)		1.36	0.92–1.99	0.12
	3–5 times	34.5 (80)	65.5 (152)		1.47	0.97–2.23	0.071
	>5 times	29.3 (71)	70.7 (171)		1.86	1.22–2.83	0.004
Admission to hospital	No ^#^	35.3 (295)	64.7 (541)	0.64	1		
	Yes	37.2 (61)	62.8 (103)		0.92	0.65–1.30	0.64
Chronic disease	No ^#^	39.6 (197)	60.4 (301)	0.008	1		
	1 disease	31.2 (93)	68.8 (205)		1.44	1.07–1.96	0.018
	>1 disease	28.7 (51)	71.3 (127)		1.63	1.12–2.36	0.010
Self-assessment of health status	Unsatisfactory ^#^	29.6 (29)	70.4 (69)	0.009	1		
	Satisfactory	29.4 (70)	70.6 (168)		1.01	0.60–1.69	0.97
	Good	36.4 (163)	63.6 (285)		0.74	0.46–1.18	0.20
	Very good or perfect	43.5 (94)	56.5 (122)		0.55	0.33–0.91	0.020
Internet use	No ^#^	25.2 (38)	74.8 (113)	0.004	1		
	Yes	37.5 (318)	62.5 (531)		0.56	0.38–0.83	0.004
The use of mobile telephony	Nonuser ^#^	23.0 (17)	77.0 (57)	0.013	1		
	Mobile phone but not a smartphone	32.4 (92)	67.6 (192)		0.62	0.34–1.13	0.12
	Smartphone	38.5 (247)	61.5 (395)		0.48	0.27–0.84	0.010
Age ^$^		41.67 (16.18)	48.20 (15.68)	<0.001	1.03	1.02–1.04	<0.001
HL score ^$^		12.70 (3.24)	13.15 (3.03)	0.046	1.05	1.001–1.10	0.046
eHL score ^$^		27.94 (5.95)	27.60 (6.43)	0.89	0.99	0.97–1.01	0.42

* *p*-value for chi2 test in case of categorical variables, for the Student’s *t*-test in case of age, and U Mann–Whitney test in case of health literacy (HL) and eHealth literacy (eHL) scores; ^&^ *p*-value for univariate logistic regression; ^$^ for age, HL and eHL scores—mean (standard deviation) was provided depending on the category of the opinion about vaccinations; ^#^ reference category of the independent variable in the logistic regression model.

**Table 3:** The determinant of the opinion about the introduction of the sugar tax.

Independent Variable	Categories of the Independent Variable	Acceptance of the Introduction of the Sugar Tax		*p* *	OR	95% CI	*p* ^&^
		No	Yes				
Sex	Male ^#^	62.5 (298)	37.5 (179)	0.84	1		
	Female	63.1 (330)	36.9 (193)		0.97	0.75–1.26	0.84
Education level	Lower than upper secondary ^#^	60.9 (109)	39.1 (70)	0.43	1		
	Upper secondary or postsecondary nontertiary	65.3 (284)	34.7 (151)		0.83	0.58–1.19	0.30
	Bachelor’s degree	63.9 (78)	36.1 (44)		0.88	0.55–1.41	0.59
	Masters’ degree or higher	59.5 (157)	40.5 (107)		1.06	0.72–1.56	0.76
Place of residence	Rural ^#^	59.7 (169)	40.3 (114)	0.29	1		
	Urban <20,000	64.2 (86)	35.8 (48)		0.83	0.54–1.27	0.38
	Urban from 20,000 to <100,000	65.1 (166)	34.9 (89)		0.80	0.56–1.13	0.20
	Urban from 100,000 to <200,000	57.5 (77)	42.5 (57)		1.10	0.72–1.67	0.66
	Urban from 200,000	67.0 (130)	33.0 (64)		0.73	0.50–1.07	0.11
Marital status	Single ^#^	72.8 (211)	27.2 (79)	<0.001	1		
	Widowed, divorced or separated	59.2 (77)	40.8 (53)		1.84	1.19–2.84	0.006
	Married	58.6 (340)	41.4 (240)		1.89	1.39–2.56	<0.001
Household net monthly income	≤1500 PLN ^#^	62.3 (149)	37.7 (90)	0.66	1		
	1500–2500 PLN	66.2 (151)	33.8 (77)		0.84	0.58–1.23	0.38
	>2500 PLN	61.9 (253)	38.1 (156)		1.02	0.74–1.42	0.90
	Refusal	60.5 (75)	39.5 (49)		1.08	0.69–1.69	0.73
Vocational status	Employed ^#^	63.6 (279)	36.4 (160)	0.001	1		
	Self-employed or farmer	60.7 (65)	39.3 (42)		1.13	0.73–1.74	0.59
	Retired or on a disability pension	55.4 (155)	44.6 (125)		1.41	1.04–1.91	0.029
	University or school student	81.0 (68)	19.0 (16)		0.41	0.23–0.73	0.003
	Unemployed	67.8 (61)	32.2 (29)		0.83	0.51–1.34	0.45
The use services of the health institution in the preceding 12 months	Not used ^#^	65.4 (102)	34.6 (54)	0.93	1		
	1–2 times	62.6 (219)	37.4 (131)		1.13	0.76–1.68	0.54
	3–5 times	62.9 (146)	37.1 (86)		1.11	0.73–1.70	0.62
	>5 times	62.4 (151)	37.6 (91)		1.14	0.75–1.73	0.55
Admitted to hospital	No ^#^	62.2 (520)	37.8 (316)	0.38	1		
	Yes	65.9 (108)	34.1 (56)		0.85	0.60–1.21	0.38
Chronic disease	No ^#^	64.7 (322)	35.3 (176)	0.31	1		
	1 disease	61.7 (184)	38.3 (114)		1.13	0.84–1.53	0.41
	>1 disease	58.4 (104)	41.6 (74)		1.30	0.92–1.85	0.14
Self-assessment of health status	Unsatisfactory ^#^	68.4 (67)	31.6 (31)	0.04			
	Satisfactory	55.0 (131)	45.0 (107)		1.77	1.08–2.90	0.025
	Good	61.8 (277)	38.2 (171)		1.33	0.84–2.13	0.23
	Very good or perfect	70.8 (153)	29.2 (63)		0.89	0.53–1.49	0.66
Internet use	No ^#^	53.0 (80)	47.0 (71)	0.007	1		
	Yes	64.5 (548)	35.5 (301)		0.62	0.44–0.88	0.007
The use of mobile telephony	Nonuser ^#^	55.4 (41)	44.6 (33)	0.034	1		
	Mobile phone but not a smartphone	58.1 (165)	41.9 (119)		0.90	0.54–1.50	0.68
	Smartphone	65.7 (422)	34.3 (220)		0.65	0.40–1.05	0.080
Age ^$^		44.20 (16.02)	48.70 (16.01)	<0.001	1.02	1.01–1.03	<0.001
HL score ^$^		12.74 (3.27)	13.41 (2.77)	0.005	1.08	1.03–1.13	0.003
eHL score ^$^		27.85 (6.71)	27.65 (5.99)	0.16	1.01	0.99–1.03	0.62

* *p*-value for chi2 test in case of categorical variables, for the Student’s *t*-test in case of age, and U Mann-Whitney test in case of HL and eHL scores; ^&^ *p*-value for univariate logistic regression; ^$^ for age, HL and eHL scores-mean (standard deviation) was provided depending on the category of the opinion about vaccinations; ^#^ reference category of the independent variable in the logistic regression model.
